# Simulation of Runoff Hydrograph on Soil Surfaces with Different Microtopography Using a Travel Time Method at the Plot Scale

**DOI:** 10.1371/journal.pone.0130794

**Published:** 2015-06-23

**Authors:** Longshan Zhao, Faqi Wu

**Affiliations:** 1 College of Natural Resources and Environment, Northwest A&F University, Yangling, Shaanxi, China; 2 College of Forestry, Guizhou University, Guiyang, Guizhou, China; 3 USDA-ARS National Soil Erosion Research Laboratory, 275 South Russell Street, West Lafayette, Indiana, United States of America; University of Aveiro, PORTUGAL

## Abstract

In this study, a simple travel time-based runoff model was proposed to simulate a runoff hydrograph on soil surfaces with different microtopographies. Three main parameters, i.e., rainfall intensity (*I*), mean flow velocity (*v*_m_) and ponding time of depression (*t*_p_), were inputted into this model. The soil surface was divided into numerous grid cells, and the flow length of each grid cell (*l*_i_) was then calculated from a digital elevation model (DEM). The flow velocity in each grid cell (*v*_i_) was derived from the upstream flow accumulation area using *v*_m_. The total flow travel time through each grid cell to the surface outlet was the sum of the sum of flow travel times along the flow path (i.e., the sum of *l*_i_/*v*_i_) and *t*_p_. The runoff rate at the slope outlet for each respective travel time was estimated by finding the sum of the rain rate from all contributing cells for all time intervals. The results show positive agreement between the measured and predicted runoff hydrographs.

## Introduction

Surface runoff is an important agent of soil detachment and sediment transportation on hillslopes [1]. Because of the distinct advantage modelling methods provided in anticipating runoff and expanding our understanding of the process and magnitude of soil erosion, researchers frequently consider how to accurately estimate the process of runoff generation and the change in runoff hydrographs over rainfall time using numerical models [2,3]. Advances have been made in the past decades, and a number of runoff and erosion models have been developed, including the well-known WEEP, EUROSEM and KINEROS models.

Runoff simulation is difficult work. First, in the natural environment, runoff generation on hillslopes is a complex process highly affected by many factors, such as soil infiltration, rainfall quantity and timing, and slope and soil properties [4–7]. Soil surface roughness is another important factor impacting surface runoff generation because it affects depression storage [4]. In the past several decades, the effect of depression storage on surface runoff has been considered in many laboratory experiments. However, these studies did not take into account the effect of soil surface roughness on depression storage. Second, surface runoff characteristics vary greatly over both space and time. As a result, it is difficult for researchers to select a number of reasonable indictors to build a model to simulate runoff generation.

Travel time of runoff spent on the soil surface is an important indicator of the quantification of the runoff process. There appears to have been an increase in runoff routing in more recent works [8–11]. Olivera and Maidment (1999), Liu et al. (2003) and Melesse and Graham (2004) [9,10,12] successively proposed individual travel time-based models for runoff routing at the watershed scale. In these models, the travel time of runoff water was equal to the flow length divide by the flow velocity, and the runoff hydrograph was then computed by convolving the area of contribution with the incremental travel time. Compared to the traditional model based on the theory of the unit hydrograph, travel time-based models can largely reduce the error between the observed and predicted values of runoff [6,9,13,14]. However, despite the publication of a number of recent studies on travel time, fewer studies in the literature have used a travel time-based approach to simulate runoff generation at the plot scale.

Compared with the watershed, unique hydrological characteristics of the land surface at the plot scale, such as the random distribution of clods on the surface, depressions and tillage direction, are significant factors influencing flow direction and flow velocity. These factors increase the uncertainly of runoff routing along a plot’s surface; however, they are usually not needed to calculate runoff at the watershed scale. Furthermore, the real soil surface is not smooth but contains many depressions and mounds. When it rains, depressions retain water and delay runoff temporarily. This naturally breaks the connectivity of flow and hence increases the length of runoff production [15]. Meanwhile, mounds divert runoff away from their local summits, which in turn leads to the convergence of runoff water in the spaces between the mounds and hence an increase in flow velocity. Thus, runoff water may converge or diverge at any time during a rainfall event due to surface microrelief. This suggests that runoff undergoes a series of processes, such as depression filling and outflowing, flow paths merging and splitting due to barriers. All of these processes lead to an increase or decrease in flow velocity and hence affect the time of rain water outflow from the surface outlet. Therefore, the unique effects of surface microrelief on runoff should be considered separately when planning to build a simulated model for runoff routing. Fortunately, some recent studies have taken surface microrelief into account, and a few of special models have been developed using this theory. In these models, the effect of surface microtopography on runoff process was considered fully. For example, Nord and Esteves (2005) [16] developed a Plot Soil Erosion Model 2D (PSEM_2D). In PSEM_2D, the runoff is computed using the Saint-Venant equation. In contrast, Chu et al. (2013) [17] used a puddle-to-puddle model to simulate a hydrograph that accounts for surface depressions.

The objective of this study was to simulate a hydrograph of runoff on the soil surface using a travel time-based runoff model at the plot scale. The simulation results obtained from this model were compared with a series of laboratory experiment results to validate the performance of the proposed model. We chose two types of surface microrelief, i.e., the mound and the depression, as our experimental subjects.

## Methodology

When it rains, rainwater that has fallen on the soil surface must first travel some distance before reaching the surface outlet or channel. In hydrology, this distance is commonly known as flow length. The time it takes for water to travel this distance is known as travel time.

In this study, we assume that the amount of runoff generated at the surface outlet is related to the travel time of rainwater; thus, a travel time-based rainfall-runoff approach was taken to simulate the hydrograph at the surface outlet. The hydrograph maps runoff within the contributing watershed area from the most upstream reaches to a downstream point along the surface channel for a given travel time. To achieve this purpose, the soil surface was first divided into a grid of *N* equally sized cells. Here, we define the flow length of rainwater travel within cell *i* as *l*_*i*_, and the amount of time it takes rainwater to travel through cell *i* is defined as *t*_*i*_. *t*_*i*_ can be obtained by dividing *l*_*i*_ by the flow velocity *v*_*i*_ in cell *i*. For all cells in the DEM, *l*_*i*_ is not a constant; rather, it varies with flow direction along the flow path. Particularly, if the cell has a horizontal or vertical flow direction, flow length equals cell size; if the cell has a diagonal flow direction, flow length is equal to the cell size multiplied by 1.414.

The flow direction for each cell was determined using an eight-direction pour point algorithm [18]. This algorithm uses a three-by-three window to determine the flow direction for the grid cell at the center of the window and finally chooses the direction of the steepest descent from among eight permitted choices. So far, this algorithm is the most popular method for investigating catchment properties, such as the extraction of river networks and boundaries of sub-watersheds. Once the pour point algorithm identifies the flow direction of each cell, a unique cell-to-cell flow path is determined from a given cell to the surface outlet.

### Calculation of travel time

Based on the definition above, the *t*_*i*_ of cell *i* can be calculated by the following equation:

$$t_{i}=l_{i}/v_{i}$$ (1)

where *t*_*i*_ is the travel time through cell *i*, s; *l*_*i*_ is the flow length of cell *i*, m; *v*_*i*_ is the flow velocity of rainwater at cell *i*, m/s. We assume that there exists a positive relationship between the flow velocity at cell *i* and the ratio of upslope accumulated flow area of cell *i* to mean accumulated flow area of all cells. Thus, the *v*_*i*_ can be calculated by the following equation:

$$v_{i}=v_{m}\frac{FA_{i}}{FA_{m}}$$ (2)

where *v*_*m*_ is the mean flow velocity of rainwater through all cells, m/s; *FA*_*i*_ is the upslope accumulated flow area of cell *i*, m^2^; *FA*_*m*_ is the mean of the accumulated flow area of all cells, m^2^. Using Eq (2), a flow velocity cell map, in which the flow velocity of each cell depends on the variable upslope accumulated flow area, was obtained. Due to the impact of local microtopography, the flow velocity is non-constant for all cells. That is, the flow velocity cell map has a non-uniform spatial distribution pattern. This is the greatest difference and largest improvement between this and previous studies, where, in general, flow velocity was considered constant.

The time needed for rainwater to move from cell *i* to the surface outlet is determined by summing the accumulated *t*_*i*_ along the flow path, that is,

$$T_{i}=\sum_{j=1}^{n}t_{i}\left(j\right)$$ (3)

where *T*_*i*_ is the accumulated time it takes for rainwater to move from cell *i* to surface outlet, s; *t*_*i*_(*j*) is the travel time through cell *j* located on the flow path, s; and *n* is the number of cells.

Previous studies have suggested that surface depressions can enhance the retention of rainwater and hence delay the initiation of surface runoff to a surface outlet [4,19]. Thus, most of rainwater is retained in the depression area at the beginning of the rain event and cannot move or contribute to runoff generation until the surface depression is completely filled by rainwater. In this case, the travel time of rainwater across the depressed area consist of two time periods: the first period is the ponding time of the depression area; and the second period is the accumulated travel time from the cell to the surface outlet (i.e., *T*_*i*_). Assuming *t*_*p*_ (s) is the ponding time of the depression, Eq (3) can be replaced by the following equation:

$${\bar{T}}_{i}=t_{p}+T_{i}$$ (4)

where ${\bar{T}}_{i}$ is the real travel time of rainwater moving from starting cell *i* to the surface outlet, s. For a given surface, the initial storage capacity of each depression is constant. Therefore, if the effect of soil infiltration on runoff is neglected, the ponding time for the depression is determined solely by the rainfall. Thus, *t*_*p*_ can be calculated as follows:

$$t_{p}=D/I$$ (5)

where *D* is the depth of the depression (mm); and *I* is the rainfall intensity (mm/h).

In accordance with Eq (4), ${\bar{T}}_{i}$ can be measured for each cell. The spatial shape of flow paths over a soil surface is generally not homogenous line pattern, but has many bends and branches due to the effect of surface microtopography. Therefore, ${\bar{T}}_{i}$ may vary from cell to cell. In addition, for flat and mound surfaces in this study, the value of *t*_*p*_ equals zero.

### Simulation of runoff hydrograph

Based on the discussion above, the real travel time ${\bar{T}}_{i}$ of water through all cells is measured. Assuming all cells have the same ${\bar{T}}_{i}$ will have the same effect on runoff quantity at the outlet. That is, rainwater that falls in cells with the same ${\bar{T}}_{i}$ will reach the surface channel at the same time. Therefore, the instantaneous runoff rate at the surface outlet can be estimated from the total rainwater falling in cells with the same ${\bar{T}}_{i}$. Consequently, the area of contributing cells with the same ${\bar{T}}_{i}$ could be obtained and the runoff rate at the surface outlet estimated by finding the sum of the rainwaters within contributing cells at each respective travel time during the rain event. The equation is

$$Q(t)=\sum_{\Delta t=0}^{T}A_{\Delta t}I$$ (6)

where *Q*(*t*) is runoff rate at the outlet (g/s); *A*_Δ*t*_ is the area of the contributing cells at each respective travel time (Δt) during rainfall (m^2^); and *I* is the rainfall intensity (mm/h). The water density used to calculate runoff rate with a unit of g/s was 1.0 g/cm^3^ in this study.

**Fig 1:**
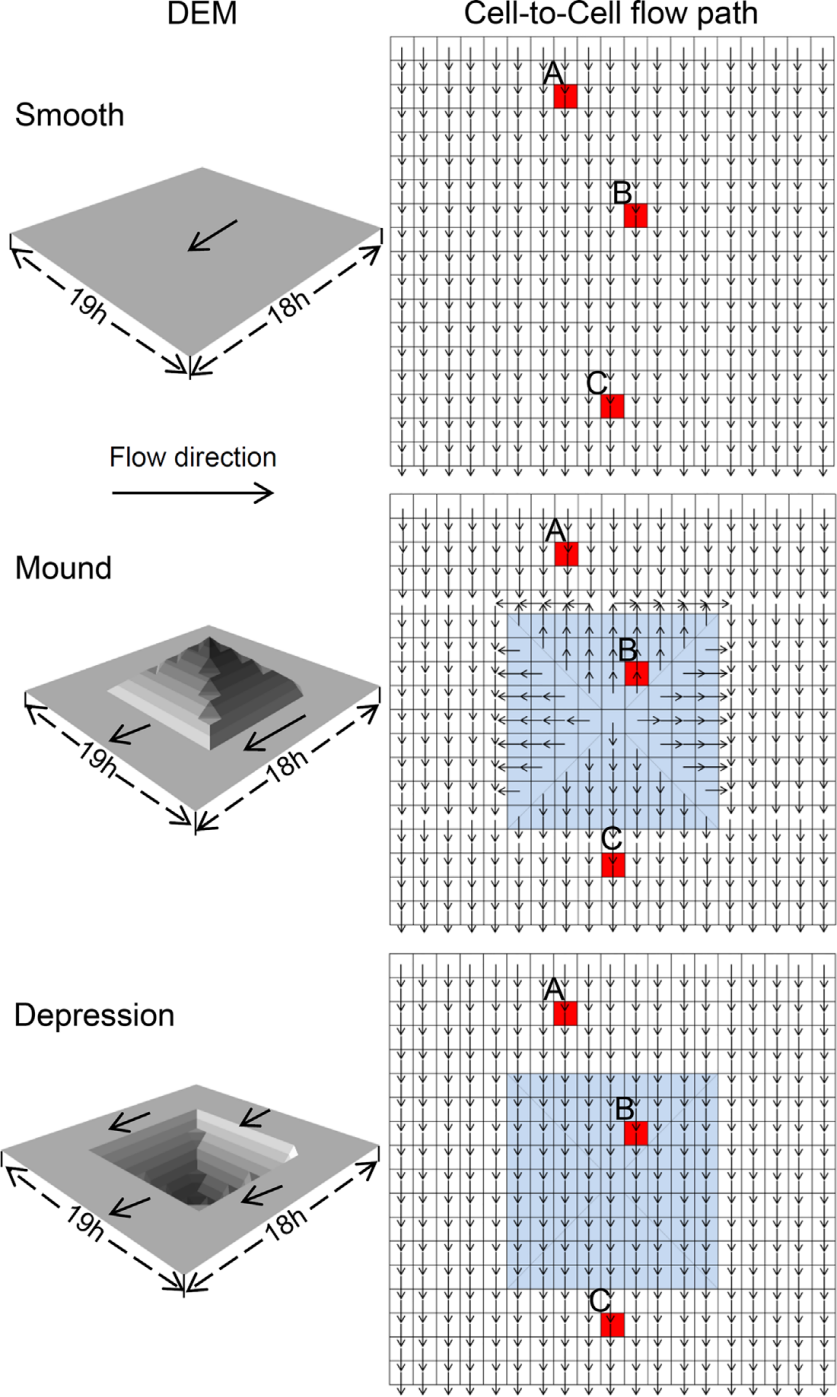
A simplified graphic of the DEM and the cell-to-cell flow path of cells.

We illustrate this approach through an example. Fig 1 shows three simplified DEMs with different microtopography structures and flow directions for each cell. Mounds and depressions were simulated using simplified geometrical shapes, similar to positive and reverse pyramids, respectively. Each DEM has 342 equally sized cells. The black line with the arrow indicates the flow direction. Cells A, B and C are three arbitrary cells in the DEM. We assume that the flow velocity for all cells is constant and rainwater can cross a cell within each time interval (Δt). In this case, the travel times of rainwater through cells A, B and C are 16Δt, 11Δt and 3Δt for the flat surface and 18Δt, 21Δt and 3Δt for the mound surface. For the depressed surface trial, travel times are 16Δt+t_p_, 11Δt+*t*_p_ and 3Δt, respectively, where *t*_p_ is the ponding time of the depression. On the basis of this rule, all cells have a corresponding time period that represents its travel time. Once we obtain the travel time of all cells, the contributing area of runoff to the surface outlet is computed by multiplying the number of cells with the same travel time and the square of the cell size.

**Fig 2:**
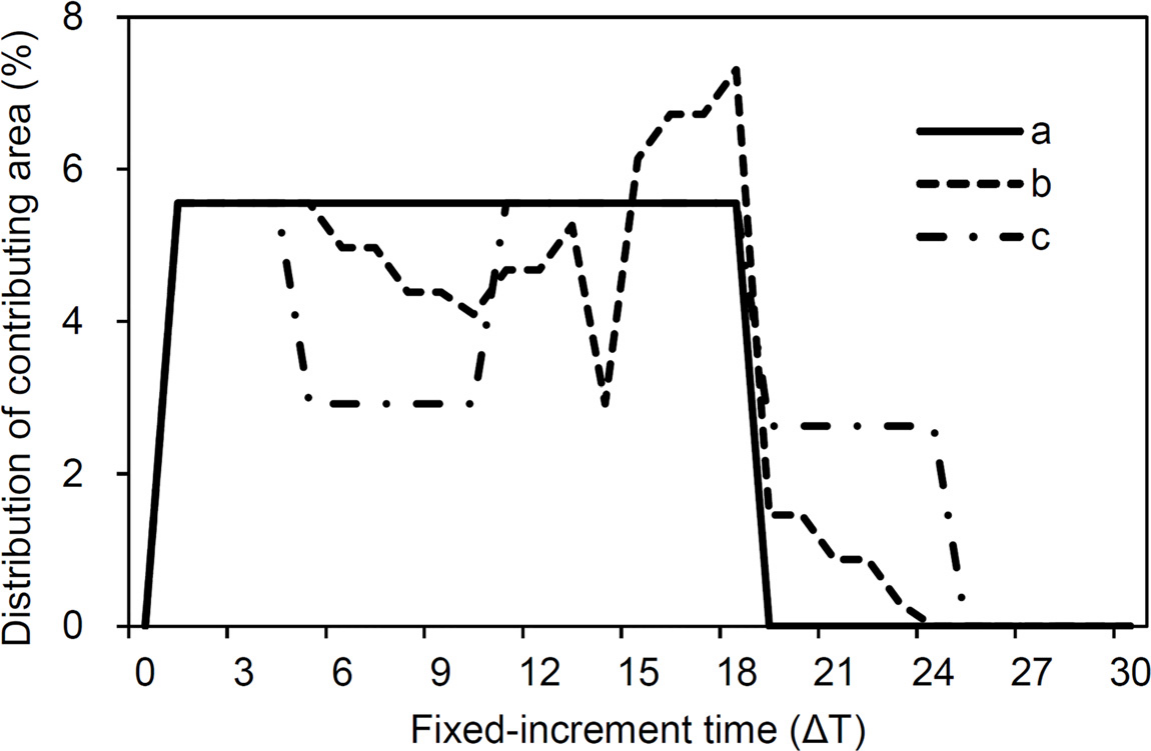
Change in runoff contribution area as time progresses when *t*_p_ = 6Δt for smooth (a), mound (b) and depression (c) (Δt is the fixed-increment time).

**Fig 3:**
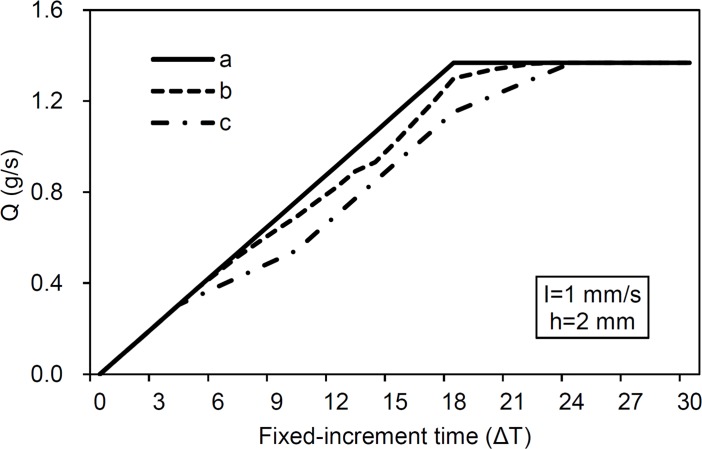
Change in runoff rate as time progresses when *t*_*p*_ = 6Δt; (a) smooth DEM, (b) mound DEM and (c) depression DEM (Δt is the fixed-increment time; *I* is rainfall intensity; *h* is the size of grid).

Fig 2 shows the change in contribution area to the surface outlet with increased travel time. It is clear that the runoff contribution areas for smooth, mounded and depressed surfaces follow different patterns of change as time progresses. Patterns differences among all three surface types appeared after 5Δt, i.e., greater fluctuations in the runoff contribution area and a longer time range for the contributing area for depressed and mounded surfaces than smooth. The reason for this difference is ascribed to how depressions and mounds affect the runoff generating process and is the theoretical foundation of our method of generating runoff hydrograph simulation. The hydrograph of runoff from three different surface forms is shown in Fig 3. It is clear that depressions and mounds both cause a reduction in the runoff rate during the rising period of the hydrograph. This difference in runoff rate between smooth and mounded/depressed surfaces is in agreement with our initial assumption, i.e., mounds and depressions affect the travel time of rainwater as it moves from a falling position to an outlet by affecting the surface flow path in different ways.

### Model evaluation

The model efficiency coefficient (*EF*) and determination coefficient (*R* ^2^) of the linear regression between simulated and measured values, and the absolute errors of the time to a steady runoff state (*ET*) and the mean steady runoff rates (*EMSR*), were used in this study to evaluate the performance of the model. The *EF* is calculated using the equation:

$$EF=1-\frac{\sum_{i=1}^{N}{\left(Q_{i}-Q_{i}^{\prime }\right)}^{2}}{\sum_{i=1}^{N}{\left(Q_{i}-\bar{Q}\right)}^{2}}$$ (7)

where *Q*_*i*_ is the measured value at discrete time *i*; $Q_{i}^{\prime }$ is the predicted value at discrete time *i*; and $\bar{Q}$ is the mean of the measured values over all times. Obviously, the bigger the *EF* is, the better the efficiency of the model performance; and the closer *R* ^2^ is to 1, the higher the prediction quality.

## Model Verification

### Runoff plot experiments

**Fig 4:**
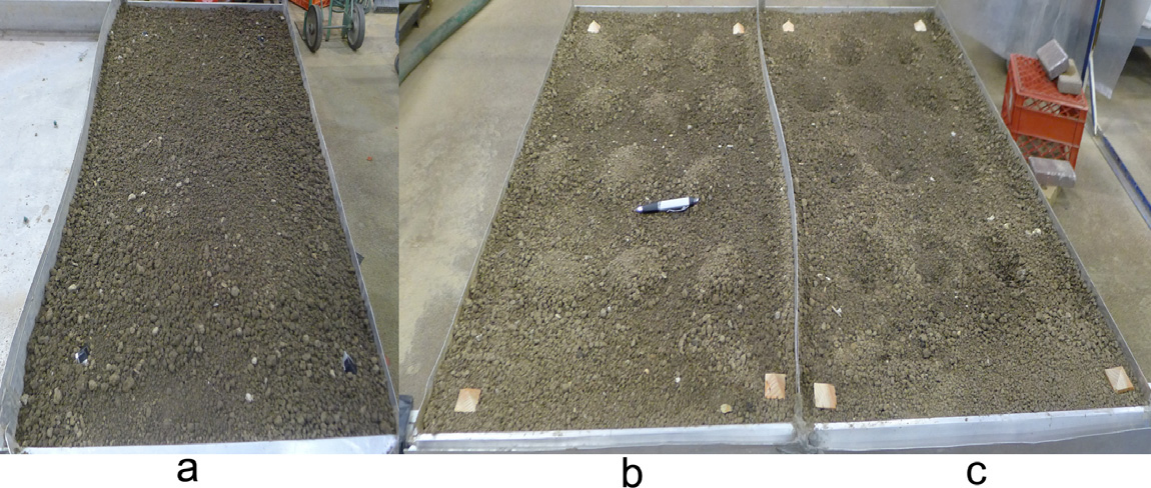
Photograph of surface microtopography with a smooth surface (a), mounds (b) and depressions (c).

The laboratory experiments contained three types of surface microtopographies, i.e., mound, depression and smooth surface (Fig 4). The soil box is 1.2 m long and 1.2 m wide, divided midway into two 0.6 m wide study areas. The soil box has drainage holes at the bottom and the study was conducted under free-drainage conditions. The rainfall intensity applied was 50 mm/h. Runoff samples were collected at five-minutes intervals to develop a runoff hydrograph. Before the rain was applied, the surface microtopography was measured using an instantaneous profile laser scanner with a horizontal resolution of 1.5 mm and a vertical resolution of 0.5 mm [20]. These measured relative elevations were used to create DEMs, which were used to run the runoff model proposed in this study. The size of each grid cell for each DEM was 2 mm.

The soil was collected from the surface horizon (0–20 cm) of a Crosby-Miami complex alfisol with 20% clay, 66% silt and 14% sand at Purdue University research farm in West Lafayette, Indiana. The soil was air dried and passed through an 8 mm wire mesh screen to remove debris and to ensure the homogeneity of the aggregate size distribution. The air-dried soil was packed into the soil box with a mean bulk density of 1.3 g/cm^3^. A wooden block was used to sweep across the soil surface to obtain a smooth surface in the test area. Subsequently, nine mounds or depressions were formed in the boxes using a small shovel. Both mounds and depressions had a hemispherical shape, were 8 to 10 cm in diameter and had a height or depth of approximately 5 cm. The mounds and depressions were uniformly arranged in each box with a spacing of 25 cm in the downslope direction. In the cross-slope direction, the spacing between mounds or depressions was 15 cm. The soil box was set to a 10% slope for each rainfall simulation run. The experiment design and conditions are listed in Table 1. Rain no.1, 4 and 7 were used to calibrate the model and others were used to verify the performance of model.

**Table 1:** Laboratory experiments with 50 mm/h rain intensity.

Rain no.	Surface microtopography	Time to steady state (min)*	Steady runoff rate (g/s)
1	Smooth	50	10.49
2	Smooth	50	9.98
3	Smooth	45	10.16
4	Mound	55	9.08
5	Mound	50	9.59
6	Mound	55	9.35
7	Depression	65	8.61
8	Depression	60	9.17
9	Depression	65	8.76

*standard deviation of all runoff rates measured after this time was smaller than 0.2.

### Model calibration

Three rain events were selected for model calibration, i.e., Rain no. 1, 4 and 7. Each rain event corresponded to one of the three types of surface microtopographies. For each one, five mean flow velocities (*v*_m_) were applied, the evaluation results of which are shown in Table 2. Results indicate that, when *v*_m_ was equal to 0.075, 0.090 and 0.060 m/s for rain event no.1, 4 and 7, respectively, a minimum absolute error of the time to a steady runoff state (*ET*) could be obtained. Considering others evaluation indicators, i.e., *EF*, *R* ^2^ and *EMSR*, we selected 0.075, 0.090 and 0.060 m/s as the optimal values of calibrated parameter *v*_m_ for rain events no. 1, 4 and 7.

**Table 2:** Statistical results of runoff prediction for rain no. 1, 4 and 7 at different values of parameter *v*_m_ (Grid size = 2 mm).

Rain no.	*v*_m_ (m/s)	*EF*	*R* ^2^	*ET* (min)	*EMSR* (g/s)
1	0.060	0.605	0.967	+10	-0.727
	0.070	0.842	0.984	+5	-0.595
	0.075	0.901	0.985	0	-0.590
	0.080	0.927	0.981	-5	-0.604
	0.090	0.925	0.963	-10	-0.574
4	0.070	0.732	0.894	+15	-0.160
	0.080	0.879	0.938	+5	-0.170
	0.090	0.924	0.967	0	-0.028
	0.100	0.903	0.982	-5	-0.027
	0.110	0.842	0.984	-15	-0.015
7	0.060	0.632	0.844	+5	+0.392
	0.070	0.847	0.885	0	+0.654
	0.080	0.837	0.916	-5	+0.819
	0.090	0.705	0.940	-10	+0.925
	0.100	0.512	0.957	-10	+1.048

*v*_m_-mean flow velocity; *EF*-the model efficiency coefficient; *R* ^2^-determination coefficient; *ET*-the absolute errors of the time to a steady runoff state between measured and predicted values; *EMSR*-the absolute errors of mean steady runoff rates between measured and predicted values.

### Model validation

**Fig 5:**
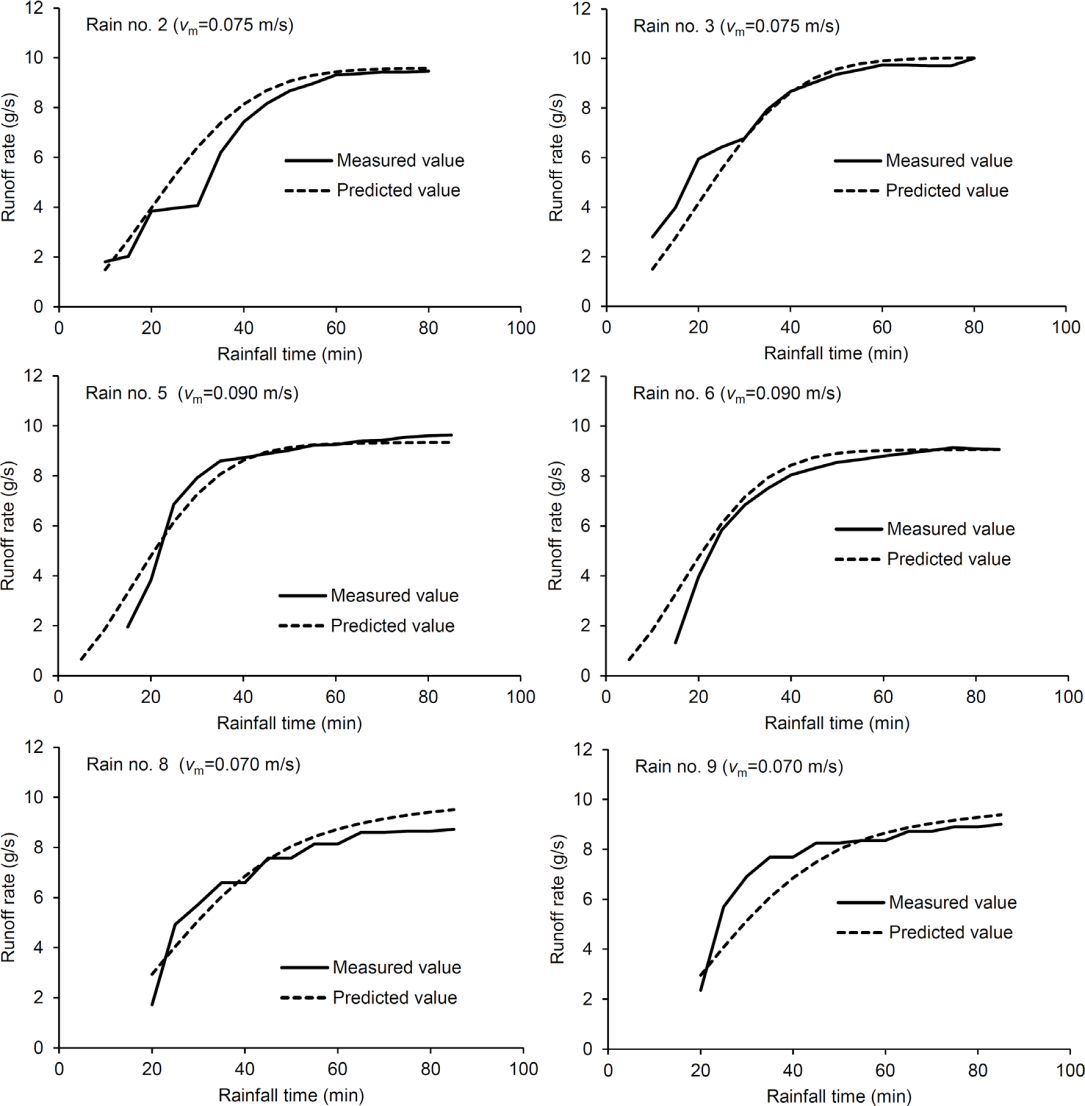
Comparison of measured and predicted runoff rates with increasing rainfall time for different surface microtopographies. (Rain no.2 and 3 for smooth surfaces; Rain no.5 and 6 for mounded surfaces; Rain no. 8 and 9 for depressed surfaces; *v*_*m*_-mean flow velocity).

Measured and predicted hydrographs for all six rain events are shown in Fig 5. Table 3 summarizes the results of the model simulation and error statistics. Five of six rain events have efficiencies greater than 0.90; all rain events have a higher determination coefficient; and the relative error of the steady runoff rates was great for rain events no. 8 and 9, followed by rain events no. 2, 5 and 3, 6. Relatively, the predicted and measured hydrographs from the mound surfaces showed the greatest similarities compared to the others when runoff reached a steady state. The predicted steady runoff rates for depressed surface were 4–7% greater than the measured values. This relatively high difference may be related to the depression’s impact on soil infiltration. In our model, the infiltration factor was neglected, so the error from the effect of infiltration was added to the estimated runoff rate. In other words, the greater the effect of infiltration on runoff process is, the higher the error between the measured and simulated values. This analysis generally agreed with the results obtained for the depressed surfaces. Several experimental studies have shown that depressions improve infiltration rates because the hydraulic conductivity of the submerged area increases as ponding pressure head increases [21–23]. Overall, the model’s predicted runoff rates were accepted by analyzing the results from four evaluation indicators.

**Table 3:** Statistical results of the model performance for different rainfall events.

Rain no.	*EF*	*R* ^2^	*ET* (min)	*EMSR* (g/s)
2	0.943	0.986	0	+0.346
3	0.911	0.948	+5	+0.260
5	0.941	0.963	-5	-0.341
6	0.923	0.982	-10	-0.112
8	0.903	0.922	+5	+0.616
9	0.807	0.834	0	+0.411

*EF*-the model efficiency coefficient; *R* ^2^-determination coefficient; *ET*-the absolute errors of the time to a steady runoff state between measured and predicted values; *EMSR*-the absolute errors of mean steady runoff rates between measured and predicted values.

### Effects of mean flow velocity, depression storage and rainfall on simulation

**Fig 6:**
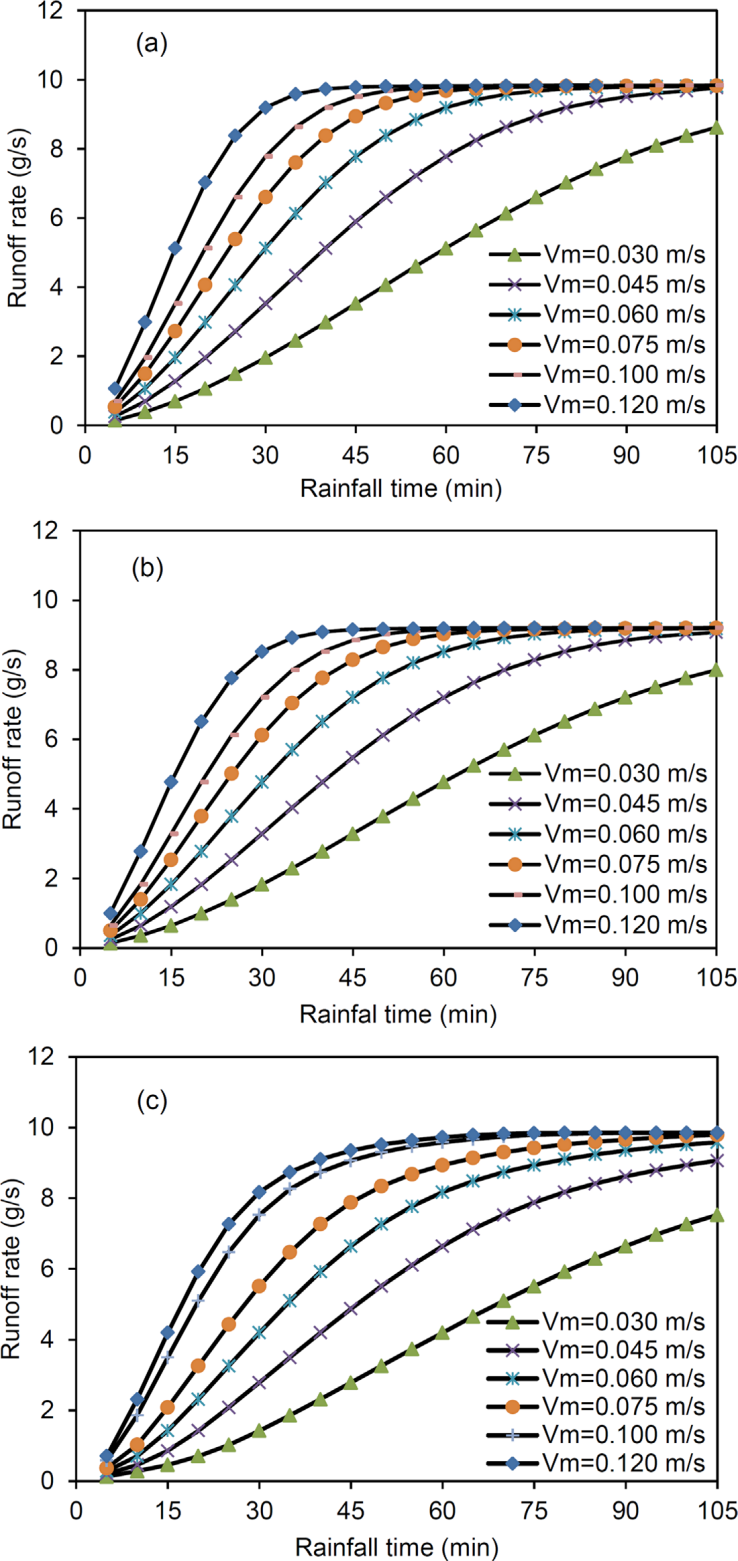
Sensitivity analysis results: model performance with a change in mean flow velocity (*v*_*m*_). (a): smooth surface; (b): mound surface; (c): depression surface.

The *v*_m_, *D* and *I* are three vital parameters influencing the performance of the model. A sensitivity analysis was conducted to assess the change in the predicted runoff rate with various parameters, i.e., *v*_m_, *D* and *I*. Changes in *v*_m_ will lead to different travel times and spatial distributions of flow velocity. These changes in turn affect the prediction result of the model. In this experiment, the location at which a steady runoff rate was achieved shifted forward, and the slope of the hydrograph during the rising period became steeper with increasing *v*_m_ over time (Fig 6).

The modelling results show that the mean flow velocity (*v*_m_) is important in controlling the features of the hydrograph. This is because the flow velocity is affected by surface microtopography and hence influences the runoff rate. Mounds increase flow velocity due to increases the gradient of the local surface, but depressions decrease flow velocity by allowing water to pond along the flow path. The travel time-runoff contribution area diagrams show the differences between mounded and depressed surfaces (Fig 2). These differences also appear in the hydrograph, where the runoff rate during the rising period is reduced and the time a steady state is reached is delayed.

**Fig 7:**
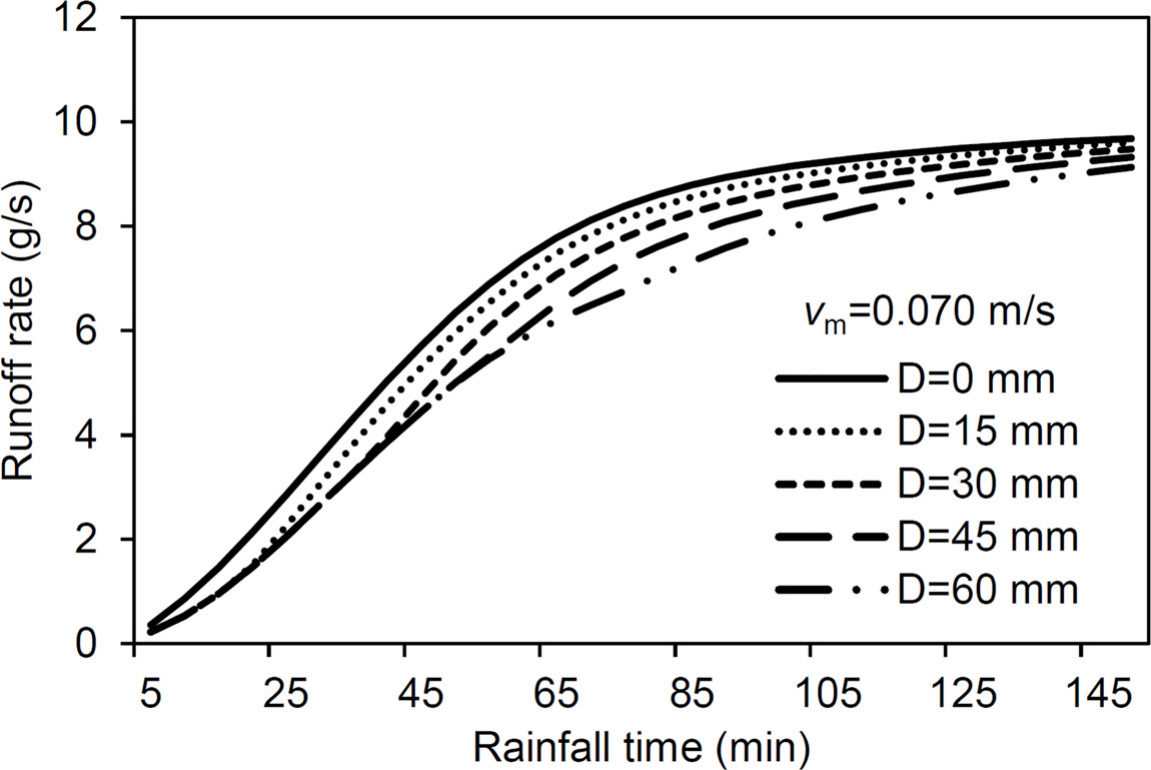
Sensitivity analysis results: model performance with a change in depression storage (D). (*v*_*m*_-mean flow velocity).

**Fig 8:**
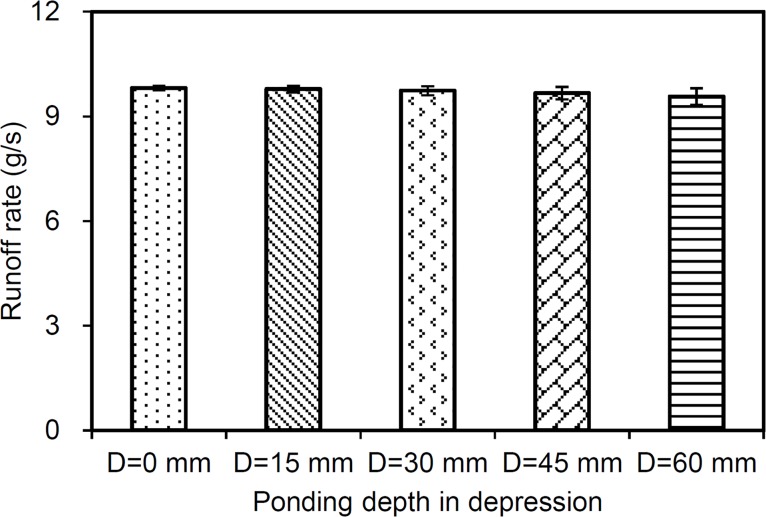
Comparison of the simulated steady runoff rate and increasing depression storage (*D*).

The effect of *D* on the simulation, as obtained from six given *D* values, is shown in Fig 7. The rate of increase along the rising limb of the hydrograph decreases with increasing *D* values. This occurred because more water was retained in depressions as *D* increased. This result supports the common conclusion in the literature that depressions reduce the runoff rates [15,24]. However, the parameter *D* has little effect on the steady runoff rate (Fig 8). This can be ascribed to the evolution of flow connectivity along the surface because the original disconnected depression surfaces are connected with increasing rain time. Darboux et al. (2002) [4] suggested that the generation of surface runoff was affected by surface roughness because of depressions filling and connecting. During the latter rain stages, flow pathways have essentially formed along the whole surface and, therefore, the effect of *D* on runoff decreases.

**Fig 9:**
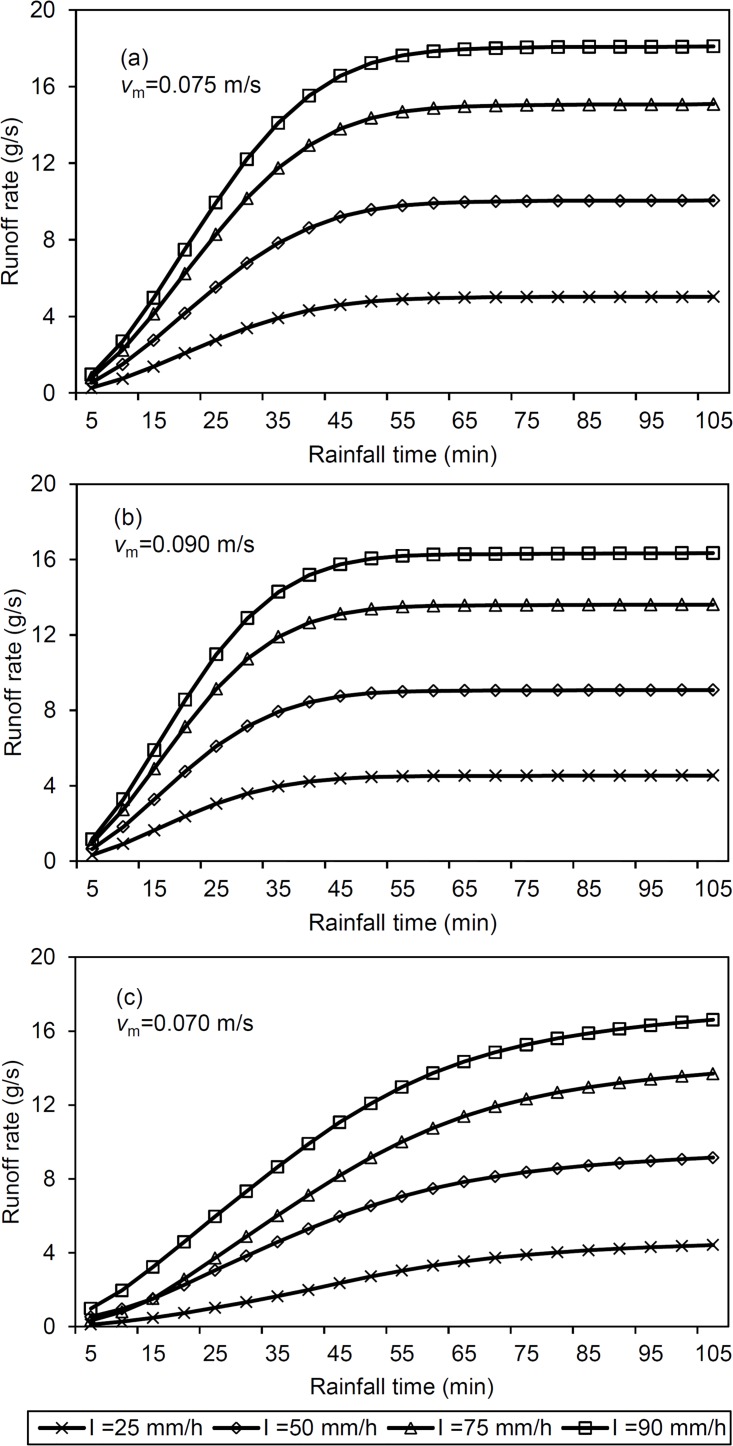
Sensitivity analysis results: model performance with a change in rainfall intensity (*I*). (a): smooth surface; (b): mound surface; (c): depression surface; *v*_m_-mean flow velocity).

The effect of *I* on the simulation is shown in Fig 9. The runoff rate increased with increasing rainfall intensity; and all hydrographs for depressed, mounded and smooth surfaces exhibit a similar shape at rain intensities of 25, 50, 75 and 100 mm/h.

## Conclusion

This study has developed a new approach to simulate hydrographs that take into account the impact of microtopography on the soil surface. The instantaneous runoff rate at the surface outlet was estimated using a spatially distributed method. The approach was applied to nine laboratory experiments for three surface microtopography structures, i.e., smooth, mounded and depressed surfaces. The estimation results aligned reasonably well with the measured results under the laboratory experiment’s conditions at the plot scale.

The advantage of this model is that it only needs three input parameters, which are the *v*_m_, *I* and *t*_p_. These three parameters are easy to obtain from a rain event. The *v*_m_ can be obtained through initial calibration; *I*, which is an essential parameter used in any runoff model, can be measured during the rainfall event, and *t*_p_ is a derived parameter that can be easily obtained from DEM data using GIS software.
